# Prevalence and risk factors of cerebral microhemorrhages and superficial siderosis in cognitively unimpaired older adults: analysis from the CHARIOT‐PRO SubStudy

**DOI:** 10.1002/alz.70594

**Published:** 2025-08-20

**Authors:** Sujin Kang, Dimitra Kafetsouli, Jamie Ford, Janice Wong, Luc Bracoud, Joyce Suhy, Parthenia Giannakopoulou, Chi Udeh‐Momoh, Tom C. Russ, Craig Ritchie, Zoi Alexopoulou, Cristian Salinas, Ziad S. Saad, Gerald Novak, Oliver Robinson, Lefkos T. Middleton

**Affiliations:** ^1^ Ageing Epidemiology (AGE) Research Unit, School of Public Health, Imperial College London, Charing Cross Hospital London UK; ^2^ Directorate of Public Health, Imperial College NHS Healthcare Trust Charing Cross Hospital Site London UK; ^3^ Johnson & Johnson, Cambridge Cambridge Massachusetts USA; ^4^ Clario Lyon France; ^5^ Clario San Mateo California USA; ^6^ School of Public Health Sciences Wake Forest University School of Medicine Winston Salem North Carolina USA; ^7^ Brain and Mind Institute Aga Khan University Nairobi Kenya; ^8^ Division of Clinical Geriatrics Centre for Alzheimer Research Karolinska Institutet Solna Sweden; ^9^ Sheffield Institute for Translational Neuroscience (SITraN) University of Sheffield, Broomhall Sheffield UK; ^10^ Division of Psychiatry, Centre for Clinical Brain Sciences University of Edinburgh Edinburgh UK; ^11^ Alzheimer Scotland Dementia Research Centre University of Edinburgh Edinburgh UK; ^12^ University of St. Andrews, North Haugh St. Andrews UK; ^13^ Scottish Brain Sciences, Scottish Gas Murrayfield Stadium Edinburgh UK; ^14^ Merck Sharp & Dohme London UK; ^15^ Takeda Pharmaceutical Company Ltd. Cambridge Massachusetts USA; ^16^ Johnson & Johnson San Diego California USA; ^17^ Johnson & Johnson Skillman New Jersey USA

**Keywords:** Alzheimer's disease, amyloid, amyloid‐related imaging abnormalities, anti‐amyloid therapies, *APOE* ε4, ARIA, CAA, cerebral microhemorrhages, cognitively unimpaired, dementia, hypercholesterolemia, hypertension, MRI, PET, superficial siderosis, white matter hyperintensities

## Abstract

**INTRODUCTION:**

Cerebral microhemorrhages (CMHs) and superficial siderosis (SS) are relatively common side effects of anti‐amyloid immunotherapies, termed amyloid‐related imaging abnormalities (ARIA‐H). They are also observed in treatment‐naïve older adults. This study explored relationships with modifiable and non‐modifiable risk factors.

**METHODS:**

This cross‐sectional study included 1414 cognitively unimpaired, treatment‐naïve individuals aged 60 to 85 years from the Cognitive Health in Ageing Register: Investigational, Observational and Trial Studies in Dementia Research (CHARIOT): Prospective Readiness cOhort (PRO) SubStudy. Relationships between CMHs/SS and cardiovascular risk factors, amyloid beta (Aβ) load, apolipoprotein E (*APOE*) ε4 status, educational attainment, and white matter hyperintensities were investigated using regression analyses and structural equation modeling.

**RESULTS:**

CMHs were observed in 8.3% of participants and SS in 1.3%. Significant risk factors for CMHs included age and hypertension. Higher education attainment appeared to have a protective effect. Elevated amyloid is a risk factor, particularly when adjusting for *APOE* ε4 status in individuals aged 70 or younger.

**DISCUSSION:**

Increasing age and hypertension are significant risk factors of CMHs. Higher educational attainment may offer a protective effect.

**Highlights:**

- Of the 1414 participants from the CHARIOT‐PRO SubStudy (CPSS), CMHs were present in 118 (8.3%), and SS was present in 18 (1.3%).
- Age and hypertension were identified as significant risk factors for CMHs, and the latter had a stronger association with the presence of CMHs among female participants. Having a bachelor's degree or higher was found to be protective.
- Elevated brain amyloid burden, particularly when adjusted for *APOE* ε4 carrier status, was identified as a risk factor in individuals aged 70 years and below.

## 1 INTRODUCTION

Cerebral microhemorrhages (CMHs) and cortical superficial siderosis (SS) appear as hemosiderin deposition on T2* weighted/gradient echo magnetic resonance imaging (MRI).^1^ CMHs are thought to represent foci of past hemorrhage into the perivascular space and surrounding vessel wall, whereas the presence of SS indicates blood permeation into the subpial or subarachnoid spaces.^2^, ^3^, ^4^ Histopathological studies have related the signal void in MRI‐detected CMHs to hemosiderin‐laden macrophages.^5^

Attention has been drawn to CMHs and SS as one of the two types of MRI abnormalities observed as adverse events of anti‐amyloid immunotherapies,^6^, ^7^, ^8^, ^9^ termed amyloid‐related imaging abnormalities (ARIA)^1^: ARIA‐H includes CMHs and SS, while ARIA‐E refers to cerebral vasogenic edema or effusion.^1^

Recent successes in trials of two anti‐amyloid immunotherapies (lecanemab and donanemab) in early clinically symptomatic Alzheimer's disease (AD) stages led to their approval in the United States and several other countries as the first disease‐modifying therapies in AD.^8^, ^9^, ^10^ Although these positive results have inspired a surge in research and development of novel disease‐modifying therapies,^11^ the occurrence of ARIA‐H and ARIA‐E has generated vigorous discussions among healthcare professionals, payers, and policymakers regarding their benefit‐to‐risk ratio, considering their effectiveness versus safety profile, in the context of the overall cost of treatment and monitoring in AD.^12^ It has been recognized that pre‐randomization, MRI‐detected CMHs are considered a risk factor for developing ARIA with anti‐amyloid beta (Aβ) monoclonal antibody therapies,^13^, ^14^, ^15^, ^16^ and the presence of four or more is considered an exclusion criterion for anti‐Aβ antibody therapy trials or use in clinical practice.^17^

The worldwide number of dementia patients among older adults is high and predicted to increase to 150 million by 2050.^18^, ^19^ A 2023 report demonstrated that dementia incidence is on the increase by as much as 25% from 2008 to 2016 in England and Wales.^20^ The high socioeconomic burden associated with AD emphasizes the urgent need for effective disease management strategies.^21^ Therefore, it is imperative to investigate the occurrence and risk factors of ARIA‐like phenomena in older adults at risk of developing AD who have not been exposed to amyloid‐targeting therapies, to better understand the natural history of these phenomena and subsequently inform mitigation strategies and future drug discovery and development.

Among treatment‐naïve patient populations (including participants at baseline or receiving a placebo in trials), the prevalence of ARIA‐E is rare (<0.8%),^2^, ^22^, ^23^, ^24^ whereas the prevalence of ARIA‐H has been reported as high as 42.5%, along the AD continuum.^2^, ^25^, ^26^

The advent of amyloid‐modifying prevention trials for cognitively unimpaired (CU) individuals with elevated Aβ load (Aβ+) or carriers of familial AD mutations^27^, ^28^, ^29^ has underscored the necessity of ascertaining the prevalence and risk factors for CMHs in relevant age groups within the general population. Prior studies including untreated CU individuals, with or without elevated cerebral Aβ status, across various age groups, have reported CMHs prevalence rates ranging from 6.5% to 39%,^30^, ^31^, ^32^, ^33^, ^34^, ^35^ illustrating the nuanced landscape of CMHs prevalence.

The Cognitive Health in Ageing Register: Investigational, Observational and Trial Studies in Dementia Research (CHARIOT): Prospective Readiness cOhort (PRO) SubStudy (denoted hereafter as CPSS) was initiated in 2015, as an industry‐sponsored study, with two sites in the United Kingdom (UK), Imperial College London (ICL) and the University of Edinburgh (EDI).^36^ The CPSS study was completed in July 2024. The CHARIOT PRO Longitudinal Study (CPLS) was initiated in March 2025, at ICL, made possible through philanthropic funding.

This publication evaluated the presence of CMHs and SS in a large cohort of CU older adults screened as part of CPSS and assessed their relationships with age, sex, Aβ load, apolipoprotein E (*APOE*) ɛ4 genotype, MRI‐detected white matter hyperintensities (WMHs), educational level, hypertension, body mass index (BMI), hypercholesterolemia, and type 2 diabetes.

## 2 METHODS

### 2.1 Study participants and design

CPSS is a prospective, longitudinal, biomarker‐enriched, observational study^36^ conducted at ICL and EDI. The study's main aims were to identify and evaluate cognitive, imaging, and fluid markers of pathology and clinical progression of AD and related dementias in older adults who were CU at screening and baseline. As an industry‐sponsored study, CPSS followed the rigorous operational model of pharmaceutical clinical trials, including regular data monitoring and validation at source.

Most participants at both centers were recruited from regional registries of healthy older adults who had consented to be invited to be considered for observational or interventional studies in AD.^37^ A total of 1914 individuals, aged 60 to 85, at ICL and 537 at EDI entered the CPSS screening process, which was completed in four consecutive separate visits within a 90‐day window and included a brain MRI scan. The major clinical exclusion criteria for the CPSS included a diagnosis of AD dementia, mild cognitive impairment (MCI), or any other degenerative brain disorder that is associated with dementia at screening or known familial autosomal dominant AD, clinical evidence of any other brain disease or other conditions leading to dementia, presence of any clinically significant unstable illness, history of stroke or transient ischemic attack (TIA), use of AD pharmacological therapies, and evidence of psychiatric/cognitive disorders/other abnormalities such as low vitamin B12 (with abnormal homocysteine and methylmalonic acid) and linked to cognitive deficits. The MRI exclusionary findings included evidence of abnormalities that could cause cognitive deficits (e.g., hydrocephalus, age‐related white matter disease of >25%, frontal or temporal atrophy not typical of AD), history or evidence of a single prior hemorrhage > 1cm^3^, multiple lacunar infarcts (two or more) or single prior infarct > 1 cm^3^, evidence of a cerebral contusion, encephalomalacia, aneurysms, vascular malformations, subdural hematoma, space‐occupying lesions, or MRI features atypical of AD dementia. Evidence of brain edema (e.g., ARIA‐E, vasogenic edema), hemosiderin deposits ≥10 mm in size, or hemosiderin deposits < 10 mm in size but > 10 in number were reviewed by the Sponsor's Medical Monitor to address plans for clinical evaluation and follow‐up as well as for potential inclusion/exclusion in the study.

**RESEARCH IN CONTEXT**

1. **Systematic review**: We reviewed the relevant literature using traditional (e.g., PubMed) sources. Given recent evidence of CMHs and SS being key side effects (ARIA‐H) of anti‐amyloid immunotherapies, it is of paramount importance to assess their prevalence and risk factors in large cohorts of cognitively unimpaired, older adults at risk of Alzheimer's disease (AD) who have not received such treatments.
2. **Interpretation**: Age, hypertension, and higher levels of cortical amyloid beta (Aβ+) – particularly when adjusted for *APOE* ε4 status – were identified as risk factors for the presence of ARIA‐H‐like MRI findings (CMHs and SS) in a population from the CPSS study. Educational attainment of a bachelor's degree or above was identified as a protective factor.
3. **Future directions**: Prospective longitudinal studies of sufficient length in more diverse populations are needed to evaluate further the risks and protective factors of CMHs and their role in the natural progression of the AD continuum and potentially provide value in the design of future clinical trials for anti‐amyloid therapies.

Furthermore, a history of first‐degree relative(s) with clinical AD was required for participants aged 60 to 65 years. This measure was added to enrich the cohort for cerebral Aβ positivity, aimed at minimizing screen failure rates. All participants who entered the CPSS longitudinal study were CU based on the Repeatable Battery for the Assessment of Neuropsychological Status (RBANS) and other cognitive tests and had a global Clinical Dementia Rating (CDR) score of 0 at screening. They were in satisfactory health and medically stable on the basis of physical and neurological examination and laboratory tests performed during the CPSS screening process.

Participants without exclusionary MRI findings were then screened for Aβ status determination via positron emission tomography (PET, *n* = 1076, including 854 at ICL and 222 at EDI) or cerebrospinal fluid (CSF) measurements (*n* = 194, including 109 at ICL and 85 at EDI). An equal number of participants with elevated (Aβ+) and matched non‐elevated (Aβ−) cerebral amyloid load were enrolled in the longitudinal study. *APOE* genotyping was initially undertaken at the early screening stages but was subsequently conducted in participants enrolled in the longitudinal CPSS study only, resulting in a sample of 833 participants with available *APOE* genotype data.

This analysis is based on the screening brain MRIs of 1414 participants (1055 at ICL and 339 at EDI). This included the prevalence of CMHs and SS and their relationship to a number of potential risk factors

### 2.2 Clinical and demographic characteristics

Medical history data, including cardiometabolic risk factors (hypertension, type 2 diabetes, and hypercholesterolemia), were collected during the CPSS screening phase, as reported by participants and later verified by their primary care physicians. Furthermore, participants presenting at screening with blood pressure measurements of 160/100 mmHg or higher, as derived from the lowest values of three repetitive measurements, were considered to have hypertension as per the UK guidelines.^38^ Height and weight were measured for BMI determination.

Other sociodemographic data collected were age, sex, race, being a resident of Greater London Region (GLR) for the ICL site and Edinburgh region (ER) for the EDI site, educational attainment (defined either as lower than a bachelor's degree or bachelor's degree and higher), and marital status.

### 2.3 MRI methodologies

MRI was performed on all eligible participants at screening. The scans were conducted based on a standardized MRI protocol, which included a 3D sagittal T1‐weighted gradient echo, two‐dimensional (2D) axial FLAIR, T2* gradient echo, dual‐echo proton‐density and T2‐weighted turbo/fast spin echo, T1‐weighted turbo/fast spin echo, and diffusion‐weighted imaging (DWI) sequences. Siemens 3T scanners were used for most subjects (Skyra 51.8%, Verio 24.4%, Prisma 12.2%, and TrioTim 11.4%). The T2* sequence settings were as follows: repetition time (TR) = 639 ms, echo time (TE) = 20 ms, slice thickness = 5 mm, slice gap = 0 mm, field of view (FOV) = 240 mm, reduced FOV = 75%, matrix = 256 × 256, averages = 1.

Three subjects (0.2%) were scanned on a General Electric Signa HDxt 1.5T scanner, and 2D sequences were acquired using 5‐mm slices with no interslice gap and an in‐plane resolution of 0.94 × 0.94 mm^2^ (1.88 × 1.88 mm^2^ for the DWI). To account for the increased sensitivity to CMHs and SS on 3T scanners compared to 1.5T, TE was increased to 30 ms at 1.5T versus 20 ms at 3T.^39^

The magnetic resonance images were assessed centrally by a single reader out of a pool of two blinded neuroradiologists at Clario (formerly Bioclinica) central laboratory to determine study eligibility. Borderline findings were reviewed by the Medical Monitor prior to final eligibility determination.

CMHs were hemosiderin deposits defined as punctate T2* hypointensities of < 10 mm in diameter. SS were hemosiderin deposits defined as linear/curvilinear T2* hypointensities, of any size.

WMH lesions were evaluated on MRI for the left and right sides of five broad brain regions (totaling 10 regions): frontal, parieto‐occipital, temporal, basal ganglia, and infratentorial/cerebellum. Each region was assigned one of four values based on visual observation according to the Age‐Related White Matter Changes (ARWMC) scale.^40^ For the basal ganglia, the following grades were available: 0 = no lesion; 1 = one focal lesion (≥5 mm); 2 = > one focal lesion (≥5 mm); and 3 = confluent lesions. For other regions, grades were defined as follows: 0 = no lesions (including symmetrical, well‐defined caps or bands); 1 = focal lesions; 2 = beginning confluence of lesions; and 3 = diffuse involvement of entire region. ARWMC scores for each region were calculated by summing left and right hemisphere scores, resulting in a range of 0 to 6.^40^

Furthermore, significant incidental cerebral abnormalities that were detected are also noted in our results.

### 2.4 Amyloid assessment

CPSS participants without exclusionary brain pathology on MRI proceeded to amyloid status assessment during the final screening stage.

Amyloid PET scans were obtained using one of three independent F18‐radiolabeled tracers: florbetapir, flutemetamol, and florbetaben. Scans were acquired in three‐dimensional (3D) mode, with correction for attenuation (computerized tomography‐based), scatter and random coincidence. They were evaluated using a hybrid visual and quantitative approach (Supplementary Text 1). Visual reads were performed by one of two neuroradiologists at Clario central laboratory, according to the prescribing information for each tracer, blinded to the standardized uptake value ratio (SUVR). The quantitative analysis involved co‐registration of the image to each participant's baseline 3D T1‐weighted MRI. A composite SUVR was calculated as the volume‐weighted average across FreeSurfer target and reference subregions derived from native‐space MRI. Positive SUVR (denoted as Aβ+) thresholds for each tracer were as follows: florbetapir: >1.14 (referenced to the whole cerebellum); florbetaben: >1.20 (referenced to cerebellar grey matter); and flutemetamol: >1.21 (referenced to the whole cerebellum). In cases where visual and quantitative analysis gave discordant results, a scan with an above‐threshold SUVR (after quality control) was always classified as positive, and a positive primary visual read (with the concurrence of a second reader) was always classified as positive if the second reader agreed.

Analysis of CSF samples included CSF Aβ_1‐42_ as the primary assay using the Fujirebio INNOTEST‐AMYLOID1‐42 and the CSF Aβ_42/40_ ratio as the confirmatory assay, using the Meso Scale Discovery (MSD) V‐plex Aβ_42/40_ ratio. Criteria for decreased Aβ_1‐42_ and Aβ_42/40_ ratios were <600 ng/L and <0.89, respectively. Participants having concordant below‐threshold values were judged to have elevated brain amyloid levels, and those with concordant above‐threshold values had non‐elevated brain amyloid levels. In case of discordant values, a second aliquot from the same sample of CSF was re‐analyzed; those with concordant values on the repeat assay were classified appropriately, while those that remained discordant between CSF assays were considered ineligible.

### 2.5 *APOE* genotyping

*APOE* ɛ4 carrier status was available for 833 participants. *APOE* genotyping was conducted using standardized procedures of extracting genomic DNA via a commercially available kit (QIAgen QIAsymphony DSP DNA Mini Kits or Promega Maxwell RSC Whole Blood DNA Kit). In this study, participants were divided into two categories: *APOE* ε4 non‐carriers (ɛ2/ɛ2, ɛ2/ɛ3, and ɛ3/ɛ3) and ε4 carriers. The latter group was further divided into heterozygous (ɛ2/ɛ4 and ɛ3/ɛ4) and homozygous (ɛ4/ɛ4) carriers.

### 2.6 Statistical analysis

CMHs prevalence was examined by age group (70 and below; 71 to 78; and 79 to 85 years), sex, primary residence (GLR or ER), Aβ status, and *APOE* ε4 carrier status. Participants were classified into (1) binary groups (0 [no CMHs] or 1+) and (2) three categorical groups (0, 1 to 3, or 4+ CMHs). SS was analyzed separately. It was found that only five cases had evidence of both CMHs and SS (0.35%).

Fisher's exact test and the Kruskal–Wallis equality‐of‐populations rank test were used to assess the significance of CMHs and SS prevalence in relation to covariates. Univariate and multivariate ordinal logistic regressions were applied using three ordinal categories of the number of CMHs (0, 1 to 3, or 4+) as the dependent variable. Cardiometabolic risk factors, amyloid status, and *APOE* ɛ4 genotype (when available), as well as sociodemographic factors, were also included in the models. Backwards elimination procedures, clinical significance, and log‐likelihood (a measure of goodness of fit) were used to select terms for the final multivariate model. Analyses were conducted on both the total study population and, separately, for those participants with available *APOE* ɛ4 genotype data and for participants from the larger sample of GLR study site. Due to their clinical relevance, subgroup analysis was performed stratifying by age group (≤70 and ≥71years) and sex. Where differences in risk factor associations with CMHs were apparent between groups, interaction analysis was conducted through the introduction of the relevant interaction term in the unstratified final multivariate model.

Structural equation modeling (SEM), a multivariate method previously demonstrated to be effective in modeling relationships between latent variables, observable indicators, and testing theories,^41^, ^42^ was employed to test hypotheses regarding the potential mediating roles of cardio‐metabolic risk factors and amyloid load in the associations between clinical and socio‐demographic characteristics and CMHs.

All statistical analyses were conducted using Stata (version 17.0, StataCorp LLC) and R statistical software (version 4.3.0, Copyright (C) 2023, The R Foundation for Statistical Computing).

## 3 RESULTS

### 3.1 Demographic information

The screening study population had a mean age of 71.1 years (SD = 5.3) and consisted of 744 females (52.6%). Participants were predominantly of white race (97.2%), and 57.1% held a bachelor's degree or a higher qualification. The mean BMI value for the total cohort was 26.2 (SD = 4.2). Among the participants who underwent amyloid‐PET or CSF analysis, 238 (18.7%) were identified as Aβ+. There were 257 (30.9%) *APOE* ε4 carriers among the 833 participants with available *APOE* ε4 genotyped data. The demographic data of participants with *APOE* genotype data were similar to the overall sample, apart from a greater prevalence of elevated amyloid burden, resulting from the selection process for genotyping (Table S1). Overall, CMHs was present in 118 (8.3%), and SS was present in 18 (1.3%) of the 1414 participants (Table 1). CMHs were more prevalent in those residing in GLR (9.4%, *p *= 0.02) and among participants over the age of 71, with 52 participants (10%) in the 71 to 78 age group and 20 (15.3%) in the 79 to 85 age group having one or more CMHs (*p *= 0.001). The presence of SS was more prevalent in men (14/70) than in women (4/774) (*p *= 0.02) (Table 2).

**TABLE 1:** Prevalence of MRI‐detected cerebral microhemorrhages and superficial siderosis for demographic and clinical characteristics.

		Cerebral microhemorrhages, *N* (%)				Superficial siderosis, *N* (%)			
Participant characteristics	*N* (%) / Mean (SD)	GLR (*N* = 1055)		ER (*N* = 359)		GLR (*N* = 1055)		ER (*N* = 359)	
		0	1+	0	1+	0	1+	0	1+
Total		956 (90.6)	99 (9.4)	340 (94.7)	19 (5.3)	1041 (98.7)	14 (1.3)	355 (98.9)	4 (1.1)
Age	71.1 (5.3)	70.9 (5.2)	73.0 (5.6)^**^	69.2 (5)	73.3 (10.3)	71.1 (5.3)	72.8 (4.9)	69.2 (5)	73.3 (10.3)
Age group									
70 and below	764 (54)	497 (93.6)	34 (6.4)	221 (94.8)	12 (5.2)	526 (99.1)	5 (0.9)	231 (99.1)	2 (0.9)
71 to 78	519 (36.7)	369 (88.7)	47 (11.3)^*^	98 (95.1)	5 (4.9)	409 (98.3)	7 (1.7)	103 (100)	–
79 to 85	131 (9.3)	90 (83.3)	18 (16.7)	21 (91.3)	2 (8.7)	106 (98.1)	2 (1.9)	21 (91.3)	2 (8.7)
Sex									
Female	744 (52.6)	504 (91.8)	45 (8.2)	186 (95.4)	9 (4.6)	546 (99.5)	3 (0.5)	194 (99.5)	1 (0.5)
Male	670 (47.4)	452 (89.3)	54 (10.7)	154 (93.9)	10 (6.1)	495 (97.8)	11 (2.2)	161 (98.2)	3 (1.8)
Race									
White	1374 (97.2)	919 (90.5)	96 (9.5)	340 (94.7)	19 (5.3)	1001 (98.6)	14 (1.4)	355 (98.9)	4 (1.1)
Others	40 (2.8)	37 (92.5)	3 (7.5)	–	–	–	–	–	–
Marital status									
Married	968 (68.5)	635 (90.5)	67 (9.5)	249 (93.6)	17 (6.4)	692 (98.6)	10 (1.4)	262 (98.5)	4 (1.5)
Others^a^	445 (31.5)	321 (91.2)	31 (8.8)	91 (97.8)	2 (2.2)	348 (98.9)	4 (1.1)	93 (100)	–
Missing	1 (0.1)	–	1 (100)	–	–	1 (100)	–	–	–
Highest education									
Less than a bachelor's degree	606 (42.9)	368 (88.9)	46 (11.1)	180 (93.8)	12 (6.3)	408 (98.6)	6 (1.4)	189 (98.4)	3 (1.6)
Bachelor's degree or higher	807 (57.1)	588 (91.7)	53 (8.3)	159 (95.8)	7 (4.2)	633 (98.8)	8 (1.2)	165 (99.4)	1 (0.6)
Missing	1 (0.1)	–	–	1 (100)	–	–	–	1 (100)	–
Amyloid status									
Non‐elevated	1032 (73)	708 (91.5)	66 (8.5)	249 (96.5)	9 (3.5)	770 (99.5)	4 (0.5)	258 (100)	–
Elevated	238 (16.8)	167 (88.4)	22 (11.6)	45 (91.8)	4 (8.2)	189 (100)	–	49 (100)	–
Missing	144 (10.2)	81 (88)	11 (12)	46 (88.5)	6 (11.5)	82 (89.1)	10 (10.9)	48 (92.3)	4 (7.7)
*APOE* ε4									
Non‐carrier	576 (40.7)	413 (89.4)	49 (10.6)	112 (98.2)	2 (1.8)	460 (99.6)	2 (0.4)	114 (100)	–
Heterozygous (ε2/ε4 or ε3/ε4)	236 (16.7)	166 (94.3)	10 (5.7)	55 (91.7)	5 (8.3)^*^	176 (100)	–	60 (100)	–
Homozygous (ε4/ε4)	21 (1.5)	13 (86.7)	2 (13.3)	5 (83.3)	1 (16.7)	14 (93.3)	1 (6.7)	5 (83.3)	1 (16.7)
Missing	581 (41.1)	364 (90.5)	38 (9.5)	168 (93.9)	11 (6.1)	391 (97.3)	11 (2.7)	176 (98.3)	3 (1.7)
Hypertension									
No	846 (59.8)	611 (92.7)	48 (7.3)	175 (93.6)	12 (6.4)	649 (98.5)	10 (1.5)	185 (98.9)	2 (1.1)
Yes	568 (40.2)	345 (87.1)	51 (12.9)^*^	165 (95.9)	7 (4.1)	392 (99)	4 (1)	170 (98.8)	2 (1.2)
Type 2 diabetes									
No	1326 (93.8)	901 (90.8)	91 (9.2)	317 (94.9)	17 (5.1)	980 (98.8)	12 (1.2)	330 (98.8)	4 (1.2)
Yes	88 (6.2)	55 (87.3)	8 (12.7)	23 (92)	2 (8)	61 (96.8)	2 (3.2)	25 (100)	–
Hypercholesterolemia									
No	923 (65.3)	597 (90.7)	61 (9.3)	251 (94.7)	14 (5.3)	648 (98.5)	10 (1.5)	262 (98.9)	3 (1.1)
Yes	491 (34.7)	359 (90.4)	38 (9.6)	89 (94.7)	5 (5.3)	393 (99)	4 (1)	93 (98.9)	1 (1.1)
BMI									
*N* = 1411^b^	26.2 (4.2)	26.2 (4.2)	26.1 (4.0)	28.0 (4.8)	4 (26.2)	26.2 (4.2)	94.6 (7.8)	28 (4.8)	26.2 (3.7)

Abbreviations: ER, Edinburgh region; GLR, Greater London Region.

^a^ Divorced/separated/single/widowed.

^b^ Three missing cases in CMHs or SS none in GLR.

^*^ *p* < 0.05; ^**^ *p* < 0.01 (Fisher's exact or two‐sample Wilcoxon rank‐sum test).

**TABLE 2:** Number of cerebral microhemorrhages and superficial siderosis by key covariates.

		Age group			Region of residence		Sex		Amyloid		*APOE* ε4		*APOE* ε4		
MRI abnormalities	*N*	70 and below	71 to 78	79 to 85	GLR	ER	Female	Male	Negative	Positive	Non‐carrier	Carrier (heterozygous/ homozygous)	Non‐carrier	Heterozygous (ε2/ ε4 or ε3/ε4)	Homozygous (ε4/ε4)
CMHs															
0	1296	718 (94)	467 (90)	111 (84.7)	956 (90.6)	340 (94.7)	690 (92.7)	606 (90.4)	957 (92.7)	212 (89.1)	525 (91.1)	239 (93)	525 (91.1)	221 (93.6)	18 (85.7)
1	69	24 (3.1)	33 (6.4)	12 (9.2)	64 (6.1)	5 (1.4)	33 (4.5)	36 (5.4)	47 (4.5)	14 (5.9)	34 (5.9)	5 (2)	34 (5.9)	5 (2.1)	0 (0)
2	22	11 (1.4)	7 (1.3)	4 (3)	16 (1.5)	6 (1.7)	10 (1.3)	12 (1.8)	15 (1.5)	7 (2.9)	11 (1.9)	6 (2.3)	11 (1.9)	5 (2.1)	1 (4.8)
3	8	3 (0.4)	4 (0.8)	1 (0.8)	7 (0.7)	1 (0.3)	4 (0.5)	4 (0.6)	5 (0.5)	1 (0.4)	2 (0.4)	1 (0.4)	2 (0.4)	1 (0.4)	0 (0)
4+	19	8 (1.1)	8 (1.5)	3 (2.3)	12 (1.1)	7 (2)	7 (1)	12 (1.8)	8 (0.8)	4 (1.7)	4 (0.7)	6 (2.3)	4 (0.7)	4 (1.7)	2 (9.5)
CMHs															
0	1296	718 (94)	467 (90)	111 (84.7)^**^	956 (90.6)	340 (94.7)^**^	690 (92.7)	606 (90.4)	957 (92.7)	212 (89.1)	525 (91.1)	239 (93)^*^	525 (91.1)	221 (93.6)	18 (85.7)^*^
1 to 3	99	38 (5)	44 (8.5)	17 (13)	87 (8.3)	12 (3.3)	47 (6.3)	52 (7.8)	67 (6.5)	22 (9.2)	47 (8.2)	12 (4.7)	47 (8.2)	11 (4.7)	1 (4.8)
4+	19	8 (1.1)	8 (1.5)	3 (2.3)	12 (1.1)	7 (2)	7 (1)	12 (1.8)	8 (0.8)	4 (1.7)	4 (0.7)	6 (2.3)	4 (0.7)	4 (1.7)	2 (9.5)
SS															
0	1396	757 (99.1)	512 (98.7)	127 (97)	1041 (98.7)	355 (98.9)	740 (99.5)	656 (97.9)^*^	1028 (99.6)	238 (100)	574 (99.7)	255 (99.2)	574 (99.7)	236 (100)	19 (90.5)^**^
1+	18	7 (0.9)	7 (1.4)	4 (3.1)	14 (1.3)	4 (1.1)	4 (0.5)	14 (2.1)	4 (0.4)	–	2 (0.4)	2 (0.8)	2 (0.4)	–	2 (9.5)

Abbreviations: CMHs, cerebral microhemorrhages; ER, Edinburgh region; GLR, Greater London Region; SS, superficial siderosis.

^*^ *p* < 0.05; ^**^ *p* < 0.01 (Fisher's exact test).

### 3.2 Prevalence of cerebral microhemorrhages and superficial siderosis with key covariates

Individuals aged 71 years or older had a higher prevalence of CMHs (*p *= 0.003) than those aged up to 70. Among the 118 participants with evidence of CMHs, a higher proportion had one to three lesions (*n* = 99, 83.9%) compared to those with four or more lesions (*n* = 19, 16.1%; *p* < 0.001; one‐sample test of proportion). Possessing at least one *APOE* ɛ4 allele was more prevalent in the CMHs 4+ group (*p *= 0.03) compared to *APOE* ɛ4 non‐carriers, particularly among homozygous cases (*p *= 0.02) (Table 2 offers a more in‐depth analysis of the severity of findings related to CMHs and SS).

### 3.3 Associations between CMHs and cardiometabolic risk factors with *APOE* and Aβ status

In univariate ordinal logistic regression models of CMHs prevalence (0, 1–3, or 4+), and without adjustment for covariates, age was identified as a risk factor for CMHs, with odds ratios (95% confidence interval [CI]) of 1.64 (1.08, 2.48) for ages 71 to 78 years and 3.07 (1.64, 5.73) for ages 79 to 85 years. In addition, the GLR group exhibited a higher prevalence of CMHs, with an odds ratio of 1.82 (1.10, 3.03), compared to the ER group. Hypertension was also significantly associated with CMHs, with an odds ratio (95% CI) of 1.49 (1.02, 2.17). Amyloid load exhibited borderline significance (*p *= 0.06; Table 3). No significant associations were found between CMHs and other cardiometabolic risk factors, including type 2 diabetes, hypercholesterolemia, and BMI (Tables S2 and S3).

**TABLE 3:** Univariate models showing risk factors for cerebral microhemorrhages. Table shows results of ordinal logistic models using an ordinal category of number of cerebral microhemorrhages (0, 1 to 3, 4+) as dependent variables and each risk factor as independent variable in separate univariate models. Results are shown separately for total population and Greater London Region (GLR) only.

	Total			GLR		
Variables	*N*	OR (95% CI)	*p* > |z|	*N*	OR (95% CI)	*p* > |z|
Age	1414	1.08 (1.04, 1.11)	**<0.001**	1055	1.08 (1.04, 1.12)	**<0.001**
Age group						
70 and below	764			531		
71 to 78	519	1.64 (1.08, 2.48)	**0.02**	416	1.61 (1.01, 2.56)	0.05
79 to 85	131	3.07 (1.64, 5.73)	**<0.001**	108	3.13 (1.6, 6.12)	**0.001**
Region of residence						
ER	359					
GLR	1055	1.82 (1.10, 3.03	**0.02**	‐	‐	‐
Sex						
Female	744			549		
Male	670	1.36 (0.93, 1.98)	0.11	506	1.35 (0.89, 2.04)	0.16
BMI (continuous)	1411	0.97 (0.93, 1.02)	0.21	1052	1 (0.95, 1.05)	0.96
BMI category						
<30	1133			877		
≥30	278	0.57 (0.33, 1)	0.05	175	0.82 (0.45, 1.48)	0.51
Education						
Less than a bachelor's degree	606			414		
Bachelor's degree or higher	807	0.75 (0.52, 1.1)	0.14	641	0.72 (0.48, 1.09)	0.12
**Amyloid**						
Non‐elevated	1032			774		
Elevated	238	1.57 (0.98, 2.52)	0.06	189	1.43 (0.86, 2.38)	0.17
*APOE* ε4						
Non‐carrier	576			462		
Carrier	257	0.79 (0.45, 1.38)	0.41	191	0.57 (0.30, 1.10)	0.10
***APOE* ε4**						
Non‐carrier	576			462		
Heterozygous (ε2/ε4 or ε3/ε4)	236	0.71 (0.39, 1.29)	0.26	176	0.51 (0.25, 1.03)	0.06
Homozygous	21	1.89 (0.53, 6.66)	0.32	15	1.51 (0.33, 6.96)	0.60
Hypertension^a^						
No	846			659		
Yes	568	1.49 (1.02, 2.17)	**0.04**	396	1.88 (1.24, 2.84)	**0.003**
Type 2 diabetes						
No	1326			992		
Yes	88	1.43 (0.72, 2.85)	0.30	63	1.44 (0.67, 3.12)	0.35
Hypercholesterolemia						
No	923			658		
Yes	491	1.08 (0.73, 1.6)	0.71	397	1.03 (0.68, 1.58)	0.88
At least one cardiometabolic risk factor						
No	706			503		
Yes	708	1.19 (0.82, 1.74)	0.36	552	1.27 (0.84, 1.93)	0.26

Abbreviations: *APOE*, apolipoprotein E; BMI, body mass index; CI, confidence interval; ER, Edinburgh region; GLR, Greater London Region; OR, odds ratio.

Bold values indicate statistical significance at *p* < 0.05.

^a^ Cardiometabolic risk factors: hypertension, type 2 diabetes, hypercholesterolemia, and BMI.

The relationship between multiple covariates and CMHs was simultaneously examined in the ordinal logistic multivariate models. Age and hypertension were identified as significant risk factors for CMHs with an odds ratio (95% CI) of 1.06 for age (1.02, 1.1). The odds of having CMHs increased by 6% for each additional year of age (assuming other variables are constant), and the odds for hypertension were 1.59 (1.03, 2.48). Additionally, a high educational attainment level of at least a bachelor's degree was identified as a protective factor, with an odds ratio (95% CI) of 0.64 (0.42, 0.98) (Table 4). In the subset with available *APOE* data. We did not observe a significant association with *APOE* ε4 carrier status in the total sample. A multivariate model without adjusting for *APOE* in the subset with available *APOE* genotyping is reported in Table S4.

**TABLE 4:** Multivariate models showing mutually adjusted risk factors for cerebral microhemorrhages. Table shows results of ordinal logistic models using an ordinal category of number cerebral microhemorrhages (0, 1 to 3, 4+) as dependent variables and all risk factors shown as independent variables in multivariable models. Results shown for total sample and Greater London Region (GLR) only and for samples with *APOE* genotyping available.

Covariates	Total			GLR		
Total	*N*	OR (95% CI), *N* = 1267	*p* > |z|	*N*	OR (95% CI), *N* = 961	*p* > |z|
Age (continuous)	1267	1.06 (1.02, 1.1)	**0.01**	961	1.07 (1.03, 1.12)	**0.002**
Sex						
Female	667			499		
Male	600	1.41 (0.92, 2.15)	0.11	462	1.33 (0.84, 2.1)	0.22
BMI (continuous)	1267	0.98 (0.93, 1.03)	0.41	961	1 (0.94, 1.05)	0.88
Region of residence						
GLR	961					
ER	306	0.45 (0.24, 0.84)	**0.01**		–	–
Education						
Less than a bachelor's degree	535			373		
Bachelor's degree or higher	732	0.64 (0.42, 0.98)	**0.04**	588	0.65 (0.41, 1.01)	0.06
Amyloid						
Non‐elevated	1030			773		
Elevated	237	1.3 (0.8, 2.11)	0.30	188	1.19 (0.7, 2.03)	0.51
Hypertension						
No	767			605		
Yes	500	1.59 (1.03, 2.48)	**0.04**	356	1.81 (1.13, 2.92)	**0.01**
Type 2 diabetes						
No	1189			903		
Yes	78	1.18 (0.53, 2.61)	0.69	58	1.19 (0.5, 2.82)	0.69
Hypercholesterolemia						
No	831			601		
Yes	436	0.8 (0.51, 1.27)	0.35	360	0.73 (0.44, 1.19)	0.21

With available *APOE* genotyping		OR (95% CI), *N* = 802	*p* > |z|		OR (95% CI), *N* = 633	*p* > |z|
Age (continuous)	802	1.05 (1, 1.1)	**0.04**	633	1.05 (1, 1.11)	0.07
Sex						
Female	416			327		
Male	386	1.51 (0.9, 2.56)	0.12	306	1.56 (0.89, 2.74)	0.12
BMI (continuous)	802	0.98 (0.92, 1.04)	0.50	633	0.99 (0.93, 1.06)	0.79
Region of residence						
GLR	633					
ER	169	0.4 (0.17, 0.92)	**0.03**		–	–
Education						
Less than a bachelor's degree	356			258		
Bachelor's degree or higher	446	0.54 (0.32, 0.91)	**0.02**	375	0.53 (0.3, 0.92)	**0.03**
Amyloid						
Non‐elevated	569			449		
Elevated	233	1.49 (0.83, 2.67)	0.19	184	1.49 (0.79, 2.78)	0.22
*APOE* ε4						
Non‐carrier	553			447		
Heterozygous (ε2/ε4 or ε3/ε4)	230	0.62 (0.32, 1.21)	0.16	172	0.47 (0.22, 1)	**0.05**
Homozygous	19	1.24 (0.26, 6.03)	0.79	14	0.66 (0.08, 5.52)	0.70
Hypertension						
No	483			389		
Yes	319	1.38 (0.8, 2.37)	0.25	244	1.79 (1, 3.19)	**0.05**
Type 2 diabetes						
No	752			594		
Yes	50	1.36 (0.53, 3.54)	0.52	39	1.17 (0.41, 3.32)	0.76
Hypercholesterolemia						
No	527			397		
Yes	275	0.75 (0.42, 1.34)	0.33	236	0.63 (0.34, 1.17)	0.14

Abbreviations: *APOE*, apolipoprotein E; BMI, body mass index; CI, confidence interval; ER: Edinburgh region; GLR, Greater London Region; OR, odds ratio.

Bold values indicate statistical significance at *p* < 0.05.

### 3.4 Stratified analysis age and sex

In the ordinal logistic multivariate models for CMHs stratified by age group (Table 5), the association with Aβ positivity showed a higher, although not statistically significant, estimated odds ratio (95% CI) of 1.89 (0.86, 4.15) in the younger group (≤70 years) compared to 1.12 (0.61, 2.06) in the older group (>70 years). In the full unstratified model, this Aβ × age group interaction was significant (*p* for interaction = 0.03). In those with available *APOE* data (*n* = 833, 58.9% of the total cohort), this difference between age groups was magnified upon adjustment for *APOE* ε4 status, with an odds ratio (95% CI) for elevated Aβ status of 3.1 (1.13, 8.53) in individuals aged ≤70 years and no association in those aged >70 years (odds ratio 1.04 (0.51, 2.13). Within this subset, without adjustment for *APOE* ε status, the odds ratio (95% CI) for amyloid positivity was 2.5 (1.03, 6.07) in individuals aged ≤70 years and 0.94 (0.48, 1.85) in those >70 years (Table S5). Our data showed a trend of increasing CMHs prevalence with age. Specifically, the percentage of individuals with one to three CMHs was 5% in those 70 years and younger to 13% in the 79 to 85 age group. Similarly, the occurrence of 4+ CMHs was 1.1% and 2.3% in these age groups.

**TABLE 5:** Multivariate models showing mutually adjusted risk factors for cerebral microhemorrhages, stratified by age and sex.

Covariates	Age 70 and younger			Age 71 or older			Female			Male		
Total	*N*	OR (95% CI), *N* = 696	*p* > |z|	*N*	OR (95% CI), *N* = 571	*p* > |z|	*N*	OR (95% CI), *N* = 667	*p* > |z|	*N*	OR (95% CI), *N* = 600	*p* > |z|
Age (continuous)		–	–		–	–	667	1.06 (1, 1.12)	0.07	600	1.05 (1, 1.11)	0.05
Sex												
Female	373			294				–	–		–	–
Male	323	1.44 (0.73, 2.84)	0.29	277	1.35 (0.78, 2.33)	0.28		–	–		–	–
BMI (continuous)	696	0.95 (0.87, 1.03)	0.22	571	0.99 (0.93, 1.06)	0.88	667	0.95 (0.89, 1.02)	0.19	600	1 (0.93, 1.08)	0.95
Region of residence												
GLR	494			467			499			462		
ER	202	0.7 (0.31, 1.57)	0.39	104	0.25 (0.09, 0.73)	**0.01**	168	0.36 (0.14, 0.95)	**0.04**	138	0.52 (0.23, 1.18)	0.12
Education												
Less than a bachelor's degree	267			268			298			237		
Bachelor's degree or higher	429	0.67 (0.34, 1.32)	0.25	303	0.63 (0.37, 1.08)	0.09	369	0.73 (0.39, 1.36)	0.32	363	0.58 (0.33, 1.03)	0.06
Amyloid												
Non‐elevated	596			434			547			483		
Elevated	100	1.89 (0.86, 4.15)	0.12	137	1.12 (0.61, 2.06)	0.72	120	1.79 (0.9, 3.58)	0.10	117	1.04 (0.52, 2.11)	0.91
Hypertension												
No	471			296			432			335		
Yes	225	1.65 (0.82, 3.35)	0.16	275	1.57 (0.89, 2.79)	0.12	235	2.44 (1.26, 4.69)	**0.01**	265	1.1 (0.6, 2)	0.77
Type 2 diabetes												
No	661			528			630			559		
Yes	35	1.6 (0.34, 7.61)	0.56	43	1.18 (0.46, 3.01)	0.73	37	1.03 (0.28, 3.84)	0.96	41	1.28 (0.46, 3.51)	0.64
Hypercholesterolemia												
No	487			344			472			359		
Yes	209	0.52 (0.22, 1.22)	0.13	227	0.98 (0.55, 1.74)	0.94	195	1.11 (0.57, 2.17)	0.77	241	0.71 (0.38, 1.31)	0.27

With available *APOE* genotyping		OR (95% CI), *N* = 430	*p* > |z|		OR (95% CI), *N* = 372	*p* > |z|		OR (95% CI), *N* = 416	*p* > |z|		OR (95% CI), *N* = 386	*p* > |z|
Age (continuous)		–	–		–	–	416	1.05 (0.98, 1.12)	0.20	386	1.05 (0.98, 1.13)	0.14
Sex												
Female	231			185				–	–		–	–
Male	199	1.25 (0.52, 3.03)	0.62	187	1.61 (0.83, 3.13)	0.16		–	–		–	–
BMI (continuous)	430	0.94 (0.84, 1.04)	0.22	372	1 (0.92, 1.08)	0.98	416	0.93 (0.85, 1.02)	0.13	386	1.02 (0.93, 1.11)	0.72
Region of residence												
GLR	325			308			327			306		
ER	105	0.35 (0.1, 1.3)	0.12	64	0.42 (0.14, 1.26)	0.12	89	0.4 (0.11, 1.41)	0.15	80	0.35 (0.11, 1.1)	0.07
Education												
Less than a bachelor's degree	179			177			196			160		
Bachelor's degree or higher	251	0.47 (0.19, 1.17)	0.11	195	0.56 (0.29, 1.07)	0.08	220	0.66 (0.3, 1.44)	0.30	226	0.47 (0.23, 0.96)	**0.04**
Amyloid												
Non‐elevated	331			238			299			270		
Elevated	99	3.1 (1.13, 8.53)	**0.03**	134	1.04 (0.51, 2.13)	0.92	117	2.29 (0.97, 5.4)	0.06	116	1.24 (0.53, 2.88)	0.62
*APOE* ε4												
Non‐carrier	283			270			291			262		
Heterozygous (ε2/ε4 or ε3/ε4)	133	0.33 (0.1, 1.1)	0.07	97	0.8 (0.35, 1.85)	0.61	116	0.66 (0.25, 1.76)	0.41	114	0.56 (0.22, 1.42)	0.22
Homozygous	14	1.72 (0.29, 10.34)	0.55	5	0 (0, NE)	0.99	9	1.69 (0.17, 16.34)	0.65	10	1 (0.11, 9.43)	0.99
Hypertension												
No	291			192			265			218		
Yes	139	1.06 (0.4, 2.78)	0.91	180	1.49 (0.75, 2.98)	0.26	151	2.01 (0.89, 4.51)	0.09	168	1.01 (0.48, 2.14)	0.98
Type 2 diabetes												
No	408			344			388			364		
Yes	22	3.31 (0.56, 19.71)	0.19	28	1.06 (0.33, 3.36)	0.93	28	1.63 (0.39, 6.74)	0.50	22	1.24 (0.33, 4.66)	0.75
Hypercholesterolemia												
No	306			221			295			232		
Yes	124	0.33 (0.09, 1.16)	0.08	151	0.91 (0.45, 1.83)	0.79	121	1.09 (0.46, 2.6)	0.84	154	0.64 (0.29, 1.4)	0.26

*Notes*: Table shows results of ordinal logistic models using an ordinal category of number of cerebral microhemorrhages (0, 1–3, 4+) as dependent variables and all risk factors shown as independent variables in multivariable models. Results shown for total sample (Greater London and Edinburgh Regions) and those with *APOE* genotyping available, stratified by age (≤70 years and >70 years) and sex.

Abbreviations: *APOE*, apolipoprotein E; BMI, body mass index; CI, confidence interval; ER, Edinburgh region; GLR, Greater London Region; OR, odds ratio; NE, not estimable due to lack of model convergence. In Table S2/S3, 0 (0, ‐) denotes the same.

Bold values indicate statistical significance at *p* < 0.05.

The association between hypertension and CMHs was stronger in women with an odds ratio (95% CI) of 2.44 (1.26, 4.69). In comparison, men had a not statistically significant odds ratio (95% CI) of 1.1 (0.6, 2). In the full unstratified model, this hypertension × sex interaction was significant (*p* for interaction = 0.03). In the models with *APOE* ε4, Aβ positivity was a borderline risk factor among females, with an odds ratio (95% CI) of 2.29 (0.97, 5.4), relative to a not statistically significant odds ratio (95% CI) of 1.24 (0.53, 2.88) among males (Table 5).

### 3.5 Path analysis

In the SEM analysis, we posited that cardiometabolic measures (hypertension, type 2 diabetes, hypercholesterolemia), BMI and Aβ status are influenced by age, sex, *APOE* ε4 status, and region of residence, which may, in turn, influence CMHs prevalence.

**FIGURE 1:**
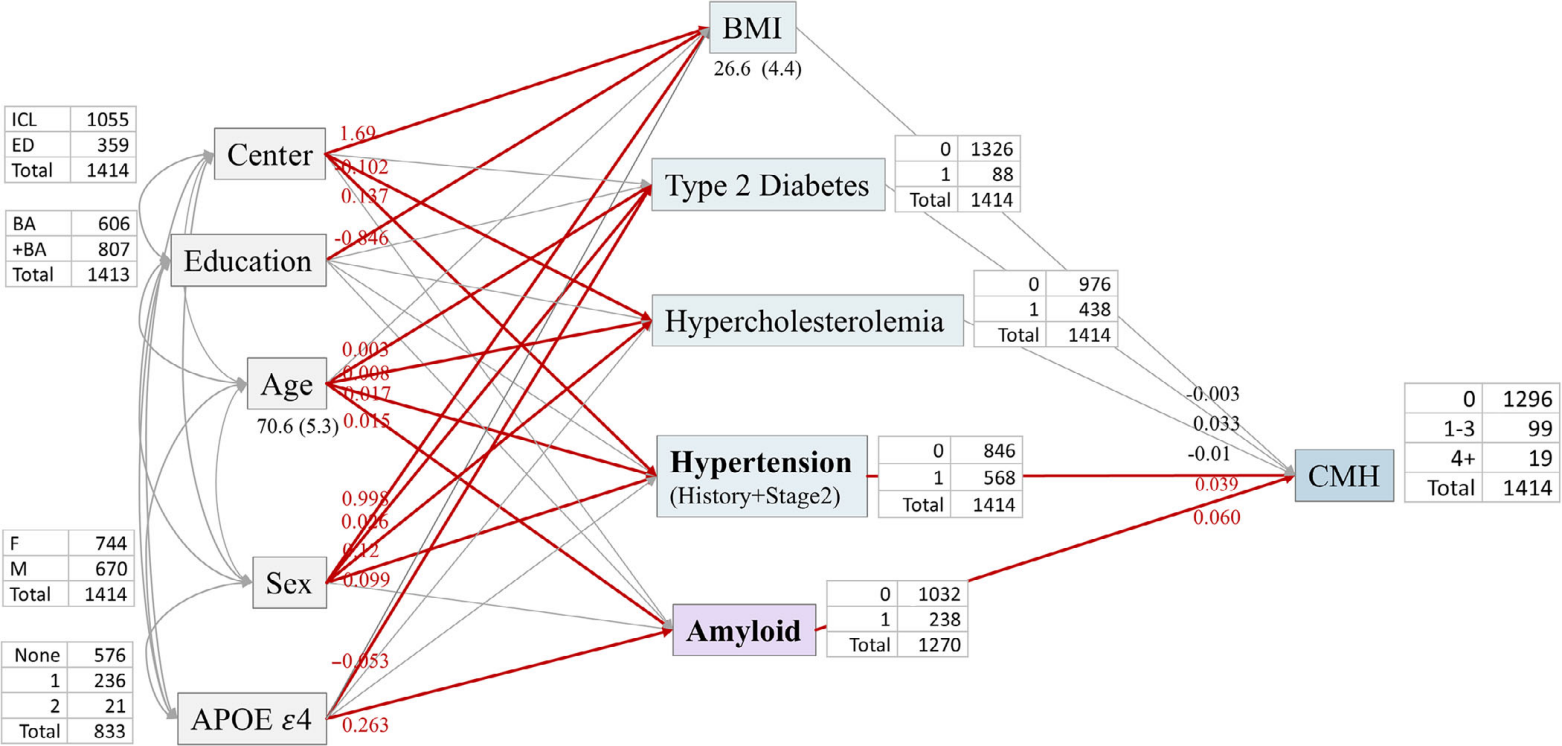
Structural equation modeling (SEM) for study population (*n* = 1414). Red arrows show statistically significant associations, with coefficients shown between variables on arrows. Goodness‐of‐fit indices were as follows: normal fit index (NFI) = 0.71; comparative fit index (CFI) = 0.70; root means square error (RMSE A) = 0.10; Akaike information criterion (AIC) = 337; (CFI) = 0.70.

To comprehensively present all pathways, a model incorporating all examined cardiometabolic risk factors was developed. The results of using maximum likelihood estimation to fit this model are shown in Figure 1. In the overall dataset, the SEM indicated the influence of *APOE* ε4 status (none, heterozygous, homozygous) and age through their effects on amyloid positivity.^43^, ^44^ The figure also highlights significant pathways involving age (β = 0.017, *p* < 0.001), sex (β = 0.099, *p* < 0.001), and region of residence (β = 0.137, *p* < 0.001) on CMHs, through their effects on hypertension (β = 0.039, *p *= 0.03). Associations between variables along pathways are shown in Figure 1 (SEM for the GLR in Figure S1).

### 3.6 White matter hyperintensities and CMHs

**FIGURE 2:**
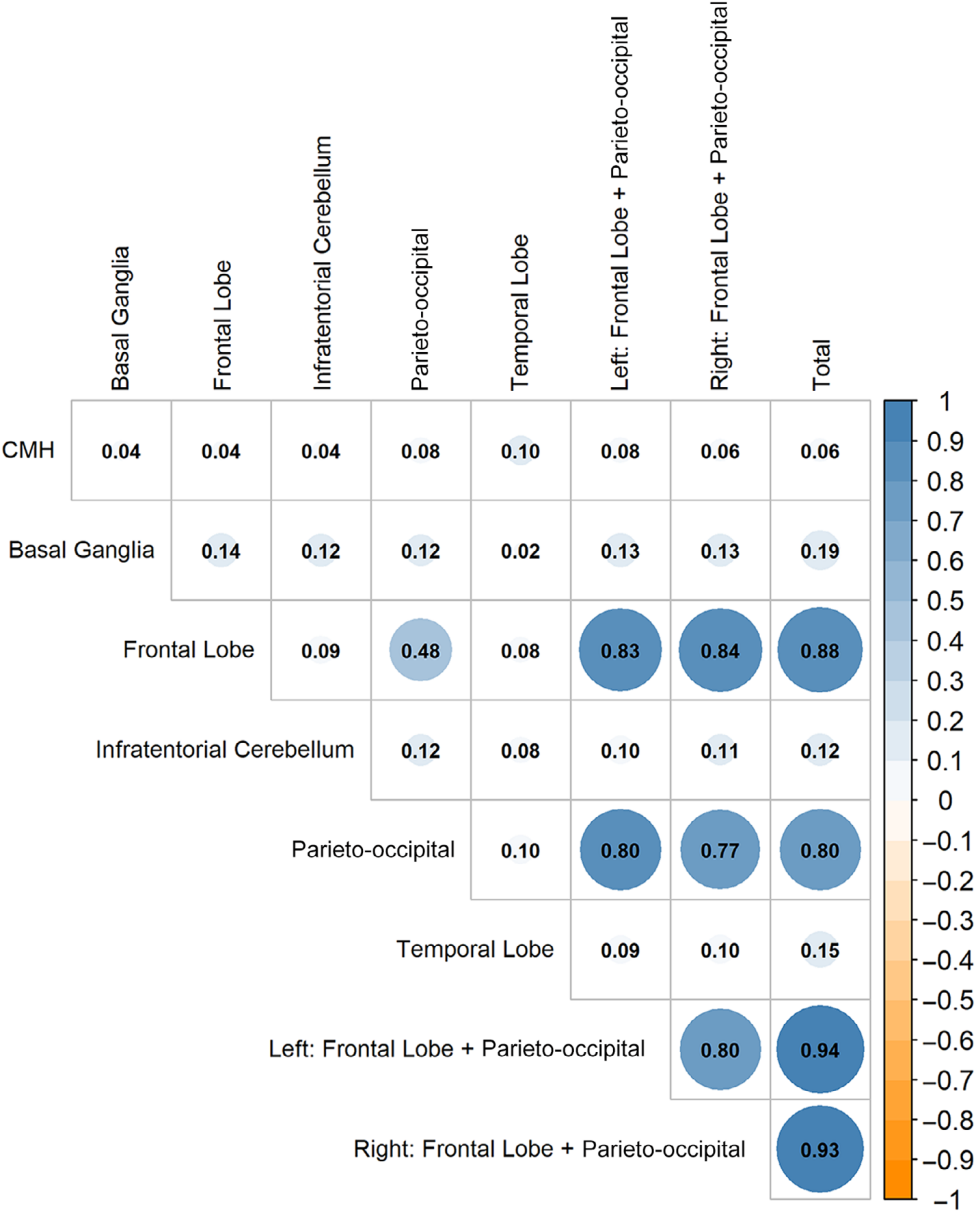
Spearman's partial rank correlation heatmap showing correlations of number of CMHs and WMHs in various brain regions (*N* = 1412). Adjusted for age, sex, BMI, region, education, *APOE* ɛ4 genotype, amyloid, hypertension, type 2 diabetes, hypercholesterolemia.

WMH have been traditionally acknowledged as markers of cerebral micro‐vascular disease,^45^ and recent reports have suggested that they may also be linked with amyloid pathologies.^46^ In the presence of CMHs (*N* = 101), the area under the receiver operating characteristic (AUROC) curve for the ARWMC rating scale in the left and right parieto‐occipital regions of WMH was 0.60 (95% CI: 0.50, 0.70), suggesting that a score of 1 or greater may help distinguish Aβ positivity. At this cutoff, the sensitivity was 61.5%, the specificity was 53.3%, and the Youden index was 0.15. For WMH in frontal lobes, the AUROC was 0.63 (95% CI: 0.53, 0.73), and a cutoff score of 2 or greater yielded similar sensitivity and specificity values (69.2% and 54.7%, respectively), with a Youden index was 0.24. In the absence of CMHs, the AUROC curve showed no significant discriminatory power (Figure S2). The parieto‐occipital region (Median IQR): 0 (0, 2) versus 1 (0, 2), *p* < 0.001; Wilcoxon rank‐sum test), as well as the left and right frontal lobe and parieto‐occipital regions showed significantly higher ARWMC rating scales in cases with CMHs present (left: 1 [0, 2] vs 1 [0, 2], *p* < 0.001; right: 1 [0, 1] vs 1 [0, 2], *p *= 0.002), (*n* = 118/1412 [8.36%]). This pattern remained consistent regardless of amyloid status. ARWMC rating scales for five regions were evaluated to assess the potential relationship between the regional distribution of WMH and the presence of CMHs, using Spearman's partial rank correlations (adjusting for age, sex, BMI, region of residence, education, *APOE* ɛ4 genotype, amyloid status, hypertension, type 2 diabetes, and hypercholesterolemia; Figure 2). The correlation magnitude between ARWMC rating scales of the frontal lobe and CMHs presence was 0.04 (*p *= 0.15), and between ARWMC rating scales of the parieto‐occipital region and CMHs presence was 0.08 (*p* < 0.01), after adjusting for all covariates. Notably, the left side of the merged frontal lobe and parieto‐occipital region showed a marginally higher correlation magnitude of 0.08 (*p* < 0.01) with CMHs presence compared to the right side (0.06; *p *= 0.04).

### 3.7 Other MRI abnormalities

Other MRI incidental abnormalities were detected in 3.9% of the scans. These included meningiomas (*n* = 12), aneurysms (*n* = 4), lacunar infarcts (*n* = 5), other infarcts (*n* = 5), encephalomalacia (*n* = 8), vascular malformations (*n* = 8), Chiari I malformations (*n* = 2), and other space‐occupying lesions (non‐malignant) (*n* = 10). However, none of these categories individually exceeded 1% of the total MRI scans (Figure S3).

## 4 DISCUSSION

In this cohort of CU adults, with a mean age of 71.1 (5.3), residing in either London or Edinburgh and screened for CPSS, the overall prevalence of CMHs was 8.3%, while SS was present in only 1.3% of participants.

Previous studies on the prevalence of CMHs showed varied results. The Austrian Stroke Prevention Study observed a 6.4% CMHs prevalence among 280 participants (mean age of 60),^47^ while the Framingham study observed a 4.7% CMHs prevalence in a subset of 472 participants with a mean age of 64.4 and 8.8% in a larger group (1965 participants) with a mean age of 65.5 years.^48^, ^49^ Furthermore, the AGES‐Reykjavik study reported a CMHs prevalence of 11.1% among participants with a mean age of 76 years.^50^ Further variability was observed in the Mayo Clinic Study of Aging, which reported a 22.6% prevalence (mean age 74.1),^51^ the updated Rotterdam study at 15.3% (mean age 60.3),^35^ the A4 study at 15.3% in 1250 Aβ+ (mean age 72.02) and the LEARN study at 8% in 538 Aβ− participants (mean age 70.53).^30^ In two multicenter studies involving participants across the AD continuum, CMHs prevalence in the CU groups was reported as 23% (mean age 73) in the ADNI study, and 21.6% (mean age 74.2) in the AIBL study.^25^, ^26^

Variability in CMHs prevalence rates across studies may stem from different population sizes and characteristics – particularly age or different selection criteria in anti‐amyloid clinical trials. The design of this industry sponsored study, including the screening exclusionary criteria, and demographic characteristics of the CU participants may account for the lower prevalence of CMHs, compared to other observational studies. Other factors affecting results may include variations in MRI methodologies, such as technical parameters of brain image acquisition and analysis, or differences in equipment, for example, magnetic field strength.

Age showed a significant association with the presence and number of CMHs, consistent with previous reports.^33^, ^35^, ^48^, ^49^, ^50^ Among the cardiometabolic risk factors, hypertension was significantly associated with CMHs presence in the total sample, in line with prior literature.^33^, ^52^, ^53^, ^54^ In our study, the association between hypertension and CMHs was marked in females.

Amyloid positivity was identified as a risk factor in individuals aged ≤70, after adjusting for *APOE* ε4 status. A multivariate subgroup analysis showed higher odds of CMHs in those aged ≤70 with amyloid positivity but not in those aged >70, although small sample sizes weakened statistical power. Interaction analyses further supported the role of amyloid positivity as a risk factor in younger individuals, with the association being more pronounced in the subset with available *APOE* genotyping and strengthened after adjustment for *APOE* ε4 status. Differences may be attributed to the higher prevalence of amyloid positivity among the participants with available *APOE* genotype data compared to the entire cohort due to their selection process. In the A4 and LEARN studies (only including Aβ+ CU participants in the former and Aβ− in the latter), the prevalence of CMHs was much higher among Aβ+ (compared to Aβ− participants), with 2% of the Aβ+ having 4 or more CMHs compared to 0% in the Aβ− group.^30^ In contrast, the Amyloid Biomarker Study group found no significant link between CMHs and amyloid pathology in CU individuals, although an age‐dependent relationship was noted in cognitively impaired patients.^55^ Previous reports also suggested that the topographic distribution of CMHs may be related to the underlying pathologies, such as cerebral amyloid angiopathy (CAA) and hypertensive vasculopathy,^5^ and that areas with high amyloid burden may be prone to CAA‐related microhemorrhages.^56^ The ARIC‐PET study demonstrated that lobar‐only CMHs are strongly associated with cortical brain amyloid load in a community‐based CU sample.^34^, ^55^ The MCSA study highlighted a strong association between global amyloid burden and CMHs odds and location, suggesting potential differences in underlying pathophysiology based on CMHs location.^33^

In our analyses, among those with *APOE* data, 18 out of 69 participants with CMHs were carriers of at least one ε4 allele, with *APOE* ε4 carrier status being slightly more prevalent in the CMHs 4+ group relative to the non‐carrier group. The relatively lower rates of *APOE* ε4 homozygosity observed within our total cohort may be related to the sample size or could be attributed to homozygous participants being more likely to meet exclusion criteria. However, the prevalence of *APOE* ε4 homozygosity (2.5%), in the genotyped group, is in line with previously reported prevalence rates.^57^ The relationship between *APOE* ε4 and CMHs is complex and varies across studies. The extended Framingham Heart study found an association between the presence of *APOE* ε4 and an increased risk of lobar CMHs.^49^ In a cohort from the Rotterdam study that underwent a follow‐up MRI scan (mean interval of 3.4 years), this association was observed primarily in *APOE* ε4 homozygous individuals.^58^, ^59^ In the same study, the younger group (average age of 60) showed a consistent association between *APOE* ε4 and CMHs.^35^ In the A4/LEARN studies, the presence of one *APOE* ε4 allele did not significantly affect CMHs presence, but *APOE* ε4 homozygosity increased the odds in the Aβ+ group.^30^ Conversely, the MCSA study found no association between *APOE* and CMHs.^33^

Based on our SEM analysis, *APOE* ε4 status may contribute to CMHs through its effect on amyloid burden. Similarly, findings from the ADNI study suggested that the risk of developing CMHs for *APOE* ε4 carriers was mediated by Aβ load.^25^ Previous reports found an association between *APOE* ε4 and increased cortical and vascular Aβ deposition, such that *APOE* ε4 is considered a risk factor for sporadic AD and CAA.^60^, ^61^ In CAA, the vascular wall integrity is compromised, creating preferential sites for developing CMHs,^56^ especially in those receiving anti‐amyloid immunotherapies.^62^ In anti‐amyloid immunotherapy clinical trials involving cognitively impaired patients in early clinical stages, the presence of the *APOE* ε4 allele was considered as a risk factor for developing both ARIA‐H and ARIA‐E.^2^, ^62^, ^63^

Our finding that a higher educational attainment level (bachelor's degree and above) has a protective effect against CMHs is intriguing. Higher educational level may represent an indicator of a more advantageous socioeconomic status, potentially leading to better living conditions and reduced exposure to harmful environmental factors. Additionally, individuals with higher educational levels are reported to engage less in unhealthy behaviors such as smoking, unhealthy diet, and lack of exercise, known contributors to cardiovascular issues.^64^ Although it has been reported that individuals with lower educational levels exhibit a higher prevalence of hypertension,^65^ we found the association between education and CMHs to be independent of hypertension and other risk factors tested. Recent data on dementia incidence trends have shown that the lower educational attainment group had a sharper increase in incidence over the last decade (2008 to 2019).^20^

It has been suggested that the spatial distribution of WMHs may reflect different etiological mechanisms underlying their occurrence, with anterior WMH potentially related to microvascular pathology, while posterior WMHs potentially related to amyloid pathology.^66^ In our study, we observed a marginally significant association between ARWMC score and CMHs in both parieto‐occipital regions and the merged left frontal and parieto‐occipital lobes. However, the small number of observed WMH lesions does not allow for a meaningful conclusion.

Recent reports suggest that cortical and parenchymal plaques primarily consist of insoluble Aβ_42_ peptide aggregates, while Aβ_40_ aggregates may form the predominant amyloid species deposited in vascular walls in CAA.^67^, ^68^, ^69^, ^70^, ^71^, ^72^

In this study, we employed multiple analytic methodologies to validate outcomes for cross‐sectional data, including univariate and multivariate regression models, and SEM. Adjustments were made for sociodemographic and clinical exposures, including Aβ and *APOE* genotyping, which may influence both the exposures and MRI outcomes.^73^, ^74^ Admittedly, statistical models often face inherent challenges to fit complex data in clinical research, and these findings warrant further studies with longitudinal follow‐up data. Furthermore, there is a need for greater harmonization of imaging methodologies and CMHs identification criteria that would enable pooled analyses and potentially reliable automated algorithms.^75^ Additionally, research focusing on the cerebral distribution patterns of CMHs and their relationship to specific Aβ species deposition could enhance understanding of ARIA genesis and associated risk factors. These may provide valuable insights into developing predictive and diagnostic tools for CMHs, enabling more precise identification of CU older adults at higher risk for developing ARIA‐H and ARIA‐E and in the discovery and development of novel anti‐amyloid agents differentially targeting Aβ disease‐related species.

Our study has several limitations. The absence of significant ethnic and cultural diversity, which is not uncommon in cohort studies of this nature, may indeed limit the generalizability of results. Future studies involving more diverse populations are warranted to confirm and extend our findings. As our analysis was cross‐sectional, the longitudinal natural history and effect of CMHs and SS over time, on cognitive and amyloid, tau, and neurodegeneration, as per the “ATN framework,” biomarker trajectories or with potential downstream cerebrovascular events were not included. Although we have observed consistency across subgroups and analyses, the multiple hypotheses tested carries the risk of false‐positive findings, and therefore caution is warranted in their interpretation.

*APOE* ε4 status was only available in 833 participants during the screening stages of the study. The rate of *APOE* ε4 homozygosity was relatively low in our CU cohort. Furthermore, we could not assess interrater reliability testing for the MRI assessments, although qualified and experienced central neuroradiologists conducted the imaging evaluations.

In conclusion, our results confirm previous research identifying increasing age and hypertension as significant risk factors for the presence of CMHs. Aβ positivity was a risk factor only among individuals aged up to 70, particularly upon adjustment for *APOE* ε4 status. Associations between hypertension and CMHs were more pronounced among women, while higher educational attainment was shown to play a protective role.

## CONFLICT OF INTEREST STATEMENT

D.K., S.K., J.F., O.R., L.B., J.S., P.G., C.U.M., and T.C.R. have no conflicts of interest to declare. L.T.M. (last on list) has received research funding (to Institution) from Johnson & Johnson, Merck, Takeda, Gates Ventures, Davos Alzheimer's Cooperative, NIHR, and UKRI. G.R. has served as a consultant for Biogen, Eisai, MSD, Actinogen, Roche, Eli Lilly, and Novo Nordisk. G.N. holds stock in Johnson & Johnson. Z.S.S. is employed by Johnson & Johnson Innovative Medicine and receives a salary and stock as compensation. J.W. is an employee of and holds stock or stock options in Janssen Pharmaceuticals. She holds stock in Biogen. Z.A. is an employee of and holds stock in Merck Sharp & Dohme. C.S. is a full‐time employee of Takeda Pharmaceuticals. Author disclosures are available in the Supporting Information.

## Supporting information



Supporting information

Supporting information
