# Supplementary material for: Prevalence and risk factors of cerebral microhemorrhages and superficial siderosis in cognitively unimpaired older adults: analysis from the CHARIOT‐PRO SubStudy
Source: Alzheimers Dement. 2025 Aug 20;21(8):e70594. doi: 10.1002/alz.70594 (PMC12365613; doi:10.1002/alz.70594)
Supplement: Supplementary file 1 — Supporting information [file ALZ-21-e70594-s002.docx]

**Supplementary Table 1** Demographic and Clinical Characteristics of Participants with and without APOE genotype data

|  |  |  | With APOE genotype data | Without APOE genotype data |
| --- | --- | --- | --- | --- |
| Total: N (%) |  | 1,414 (100) | 833 (58.91) | 581 (41.09) |
| Cerebral microhaemorrhages | 0 | 1,296 (91.65) | 764 (58.95) | 532 (41.05) |
|  | 1+ | 118 (8.35) | 69 (58.47) | 49 (41.53) |
| Age: Mean (SD) |  | 71.1 (5.3) | 70.81 (5.24) | 70.37 (5.29) |
| Age group | 70 and below | 764 (54) | 448 (58.64) | 316 (41.36) |
|  | 71-78 | 519 (36.7) | 300 (57.8) | 219 (42.2) |
|  | 79-85 | 131 (9.3) | 85 (64.89) | 46 (35.11) |
| Sex | Female | 744 (52.6) | 434 (58.33) | 310 (41.67) |
|  | Male | 670 (47.4) | 399 (59.55) | 271 (40.45) |
| Race | White | 1,374 (97.2) | 807 (58.73) | 567 (41.27) |
|  | Others | 40 (2.8) | 26 (65) | 14 (35) |
| Marital status | Married | 968 (68.5) | 565 (58.37) | 403 (41.63) |
|  | Others**^†^** | 445 (31.5) | 267 (60) | 178 (40) |
|  | Missing | 1 (0.1) | 1 (100) | - |
| Highest Education | Less than a bachelor's degree | 606 (42.9) | 370 (61.06) | 236 (38.94) |
|  | Bachelor's degree or higher | 807 (57.1) | 462 (57.25) | 345 (42.75) |
|  | Missing | 1 (0.1) | 1 (100) | - |
| Amyloid status | Non-elevated | 1,032 (73) | 571 (55.33) | 461 (44.67) |
|  | Elevated | 238 (16.8) | 234 (98.32)** | 4 (1.68) |
|  | Missing | 144 (10.2) | 28 (19.44) | 116 (80.56) |
| Hypertension | No | 846 (59.8) | 502 (59.34) | 344 (40.66) |
|  | Yes | 568 (40.2) | 331 (58.27) | 237 (41.73) |
| Type 2 Diabetes | No | 1,326 (93.8) | 782 (58.97) | 544 (41.03) |
|  | Yes | 88 (6.2) | 51 (57.95) | 37 (42.05) |
| Hypercholesterolemia | No | 923 (65.3) | 551 (59.7) | 372 (40.3) |
|  | Yes | 491 (34.7) | 282 (57.43) | 209 (42.57) |
| BMI: Mean (SD) | N=1,411*^‡^* | 26.2 (4.2) | 26.65 (4.51) | 26.61 (4.32) |
| * *p* < 0.05; ** *p* < 0.01 (Fisher's exact or Two-sample Wilcoxon rank-sum test)  † Divorced/Separated/ Single/ Widowed  *‡* Three missing cases in CMH | | | | |

**Supplementary Table 2** The ordinal logistic multivariate models for microbleeds (0, 1–3, 4+) by subgroup (**GLR only**)

|  |  |  | **Age Up to 70** |  |  | **Age 71 or older** |  |  | **Female** |  |  | **Male** |  |
| --- | --- | --- | --- | --- | --- | --- | --- | --- | --- | --- | --- | --- | --- |
| **Total** |  | N | OR (95% CI), N=494 | P>\|z | N | OR (95% CI), N=467 | P>\|z\| | N | OR (95% CI), N=499 | P>\|z\| | N | OR (95% CI), N=462 | P>\|z\| |
| Age (continuous) |  |  | - | - |  | - | - | 499 | 1.06 (1, 1.13) | 0.05 | 462 | 1.07 (1.01, 1.13) | **0.02** |
| Sex | Female | 265 |  |  | 234 |  |  |  | - | - |  | - | **-** |
|  | Male | 229 | 1.14 (0.53, 2.48) | 0.74 | 233 | 1.4 (0.79, 2.47) | 0.25 |  | - | - |  | - | **-** |
| BMI (continuous) |  | 494 | 0.98 (0.9, 1.07) | 0.67 | 467 | 1 (0.93, 1.07) | 0.98 | 499 | 0.96 (0.89, 1.04) | 0.32 | 462 | 1.03 (0.95, 1.12) | 0.47 |
| Education | Less than a bachelor’s degree | 163 |  |  | 210 |  |  | 211 |  |  | 162 |  |  |
|  | Bachelor's degree or higher | 331 | 0.64 (0.3, 1.4) | 0.27 | 257 | 0.63 (0.36, 1.11) | 0.11 | 288 | 0.79 (0.41, 1.54) | 0.49 | 300 | 0.53 (0.29, 0.99) | **0.05** |
| Amyloid | Non-elevated | 419 |  |  | 354 |  |  | 405 |  |  | 368 |  |  |
|  | Elevated | 75 | 1.82 (0.74, 4.48) | 0.19 | 113 | 1.06 (0.55, 2.01) | 0.87 | 94 | 1.59 (0.75, 3.35) | 0.23 | 94 | 1 (0.46, 2.16) | 0.99 |
| Hypertension | No | 354 |  |  | 251 |  |  | 335 |  |  | 270 |  |  |
|  | Yes | 140 | 1.74 (0.77, 3.91) | 0.18 | 216 | 1.9 (1.05, 3.45) | 0.04 | 164 | 2.81 (1.39, 5.66) | **0.004** | 192 | 1.21 (0.63, 2.33) | 0.57 |
| Type 2 Diabetes | No | 469 |  |  | 434 |  |  | 472 |  |  | 431 |  |  |
|  | Yes | 25 | 0.85 (0.1, 7.23) | 0.89 | 33 | 1.35 (0.52, 3.49) | 0.54 | 27 | 1.27 (0.33, 4.93) | 0.73 | 31 | 1.12 (0.36, 3.48) | 0.84 |
| Hypercholesterolemia | No | 330 |  |  | 271 |  |  | 345 |  |  | 256 |  |  |
|  | Yes | 164 | 0.59 (0.23, 1.5) | 0.27 | 196 | 0.79 (0.43, 1.45) | 0.45 | 154 | 0.87 (0.41, 1.82) | 0.71 | 206 | 0.71 (0.36, 1.39) | 0.31 |
| **With available APOE genotyping** |  | N | OR (95% CI), N=325 | P>\|z\| | N | OR (95% CI), N=308 | P>\|z\| | N | OR (95% CI), N=327 | P>\|z\| | N | OR (95% CI), N=306 | P>\|z\| |
| Age (continuous) |  |  | - | - |  | - | - | 327 | 1.05 (0.97, 1.13) | 0.24 | 306 | 1.04 (0.97, 1.13) | 0.25 |
| Sex | Female | 176 |  |  | 151 |  |  |  | - | - |  | - | - |
|  | Male | 149 | 1.11 (0.42, 2.89) | 0.84 | 157 | 1.86 (0.91, 3.8) | 0.09 |  | - | - |  | - | - |
| BMI (continuous) |  | 325 | 0.95 (0.86, 1.06) | 0.38 | 308 | 1.01 (0.93, 1.1) | 0.84 | 327 | 0.93 (0.84, 1.03) | 0.16 | 306 | 1.04 (0.94, 1.14) | 0.48 |
| Education | Less than a bachelor’s degree | 117 |  |  | 141 |  |  | 148 |  |  | 110 |  |  |
|  | Bachelor's degree or higher | 208 | 0.44 (0.17, 1.16) | 0.10 | 167 | 0.56 (0.28, 1.11) | 0.10 | 179 | 0.63 (0.28, 1.43) | 0.27 | 196 | 0.46 (0.21, 0.98) | **0.04** |
| Amyloid | Non-elevated | 251 |  |  | 198 |  |  | 236 |  |  | 213 |  |  |
|  | Elevated | 74 | 2.93 (0.99, 8.71) | **0.05** | 110 | 1.14 (0.53, 2.45) | 0.73 | 91 | 2.15 (0.85, 5.42) | 0.11 | 93 | 1.36 (0.56, 3.3) | 0.49 |
| **APOE ε4** | Non-Carrier | 214 |  |  | 233 |  |  | 236 |  |  | 211 |  |  |
|  | Heterozygous (ε2/ε4 or ε3/ε4) | 102 | 0.25 (0.06, 0.97) | **0.05** | 70 | 0.61 (0.24, 1.57) | 0.31 | 91 | 0.52 (0.17, 1.6) | 0.25 | 86 | 0.4 (0.14, 1.15) | 0.09 |
|  | Homozygous | 9 | 0.9 (0.09, 9.31) | 0.93 | 5 | 0 (0, -) | 0.98 | 236 | 3.16 (0.28, 35.16) | 0.35 | 9 | 0 (0, -) | 0.99 |
| Hypertension | No | 227 |  |  | 162 |  |  | 86 |  |  | 177 |  |  |
|  | Yes | 98 | 1.41 (0.51, 3.87) | 0.50 | 146 | 2.02 (0.96, 4.25) | 0.06 | 5 | 2.44 (1.03, 5.81) | **0.04** | 129 | 1.39 (0.63, 3.08) | 0.42 |
| Type 2 Diabetes | No | 308 |  |  | 286 |  |  | 212 |  |  | 289 |  |  |
|  | Yes | 17 | 1.12 (0.11, 11.09) | 0.92 | 22 | 1.21 (0.37, 3.95) | 0.75 | 115 | 1.97 (0.45, 8.66) | 0.37 | 17 | 0.75 (0.16, 3.61) | 0.72 |
| Hypercholesterolemia | No | 221 |  |  | 176 |  |  | 305 |  |  | 167 |  |  |
|  | Yes | 104 | 0.44 (0.13, 1.53) | 0.20 | 132 | 0.64 (0.3, 1.38) | 0.25 | 22 | 0.82 (0.31, 2.15) | 0.68 | 139 | 0.56 (0.24, 1.29) | 0.17 |
| Abbreviations: OR = odds ratio; CI = confidence interval; GLR: Greater London Region, ER: Edinburgh region | | | | | | | | | | | | | |

**Supplementary Table 3** The ordinal logistic multivariate models for microbleeds (0, 1–3, 4+) by subgroup (**ER only**)

|  |  |  | **Total** | |  | **Age Up to 70** |  |  | **Age 71 or older** |  |  | **Female** |  |  | **Male** |  |
| --- | --- | --- | --- | --- | --- | --- | --- | --- | --- | --- | --- | --- | --- | --- | --- | --- |
| **Total** |  | N | OR (95% CI), N=306 | P>\|z | N | OR (95% CI), N=202 | P>\|z | N | OR (95% CI), N=104 | P>\|z\| | N | OR (95% CI), N=168 | P>\|z\| | N | OR (95% CI), N=138 | P>\|z\| |
| Age (continuous) |  | 306 | 0.96 (0.85, 1.09) | 0.55 |  | - | - |  | - | - | 168 | 0.93 (0.76, 1.13) | 0.46 | 138 | 0.95 (0.81, 1.11) | 0.53 |
| Sex | Female | 168 |  |  | 108 |  |  | 60 |  |  |  | - | - |  | - | - |
|  | Male | 138 | 2.61 (0.77, 8.78) | 0.12 | 94 | 3.63 (0.78, 16.98) | 0.10 | 44 | 1.25 (0.13, 11.82) | 0.85 |  | - | - |  | - | - |
| BMI (continuous) |  | 306 | 0.87 (0.74, 1.03) | 0.10 | 202 | 0.83 (0.67, 1.03) | 0.09 | 104 | 0.99 (0.7, 1.38) | 0.94 | 168 | 0.88 (0.68, 1.14) | 0.34 | 138 | 0.87 (0.7, 1.08) | 0.21 |
| Education | Less than a bachelor’s degree | 162 |  |  | 104 |  |  | 58 |  |  | 87 |  |  | 75 |  |  |
|  | Bachelor's degree or higher | 144 | 0.61 (0.19, 1.98) | 0.41 | 98 | 0.76 (0.19, 3.07) | 0.70 | 46 | 0.52 (0.04, 6.5) | 0.61 | 81 | 0.21 (0.02, 2.19) | 0.19 | 63 | 0.87 (0.19, 3.96) | 0.86 |
| Amyloid | Non-elevated | 257 |  |  | 177 |  |  | 80 |  |  | 142 |  |  | 115 |  |  |
|  | Elevated | 49 | 2.63 (0.7, 9.84) | 0.15 | 25 | 2.02 (0.35, 11.81) | 0.43 | 24 | 5.5 (0.46, 65.42) | 0.18 | 26 | 3.8 (0.5, 29.12) | 0.20 | 23 | 2.67 (0.39, 18.1) | 0.31 |
| Hypertension | No | 162 |  |  | 117 |  |  | 45 |  |  | 97 |  |  | 65 |  |  |
|  | Yes | 144 | 0.72 (0.21, 2.45) | 0.60 | 85 | 1.63 (0.37, 7.19) | 0.52 | 59 | 0.15 (0.01, 2.17) | 0.17 | 71 | 0.54 (0.06, 4.59) | 0.58 | 73 | 0.7 (0.14, 3.42) | 0.66 |
| Type 2 Diabetes | No | 286 |  |  | 192 |  |  | 94 |  |  | 158 |  |  | 128 |  |  |
|  | Yes | 20 | 1.72 (0.18, 16.16) | 0.64 | 10 | 5.24 (0.41, 66.17) | 0.20 | 10 | 0 (0, -) | 0.99 | 10 | 0 (0, -) | 0.99 | 10 | 3.93 (0.34, 45.53) | 0.27 |
| Hypercholesterolemia | No | 230 |  |  | 157 |  |  | 73 |  |  | 127 |  |  | 103 |  |  |
|  | Yes | 76 | 1.37 (0.38, 4.97) | 0.63 | 45 | 0.23 (0.02, 2.65) | 0.24 | 31 | 11.29 (0.85, 150.43) | 0.07 | 41 | 9.37 (1.06, 82.98) | **0.04** | 35 | 0.31 (0.03, 3.07) | 0.32 |
| Abbreviations: OR = odds ratio; CI = confidence interval; GLR: Greater London Region, ER: Edinburgh region | | | | | | | | | | | | | | | | |

**Supplementary Table 4** The ordinal logistic multivariate models for microbleeds (0, 1–3, 4+) (In population **with available APOE genotype data**)

|  |  |  | **Total** |  |  | **GLR** |  |
| --- | --- | --- | --- | --- | --- | --- | --- |
| **With available APOE genotyping** |  | N | OR (95% CI), N=802 | P>\|z\| | N | OR (95% CI), N=633 | P>\|z\| |
| Age (continuous) |  | 802 | 1.06 (1.01, 1.11) | **0.03** | 633 | 1.06 (1, 1.11) | **0.04** |
| Sex | Female | 416 |  |  | 327 |  |  |
|  | Male | 386 | 1.52 (0.9, 2.56) | 0.12 | 306 | 1.56 (0.89, 2.72) | 0.12 |
| BMI (continuous) |  | 802 | 0.98 (0.92, 1.04) | 0.54 | 633 | 0.99 (0.93, 1.06) | 0.88 |
| Region of residence | GLR | 633 |  |  |  |  |  |
|  | ER | 169 | 0.38 (0.17, 0.88) | **0.02** | - | - | - |
| Education | Less than a bachelor’s degree | 356 |  |  | 258 |  |  |
|  | Bachelor's degree or higher | 446 | 0.54 (0.32, 0.91) | **0.02** | 375 | 0.53 (0.3, 0.92) | **0.02** |
| Amyloid | Non-elevated | 569 |  |  | 449 |  |  |
|  | Elevated | 233 | 1.32 (0.77, 2.27) | 0.32 | 184 | 1.19 (0.66, 2.14) | 0.56 |
| Hypertension | No | 483 |  |  | 389 |  |  |
|  | Yes | 319 | 1.35 (0.78, 2.32) | 0.28 | 244 | 1.75 (0.99, 3.11) | 0.06 |
| Type 2 Diabetes | No | 752 |  |  | 594 |  |  |
|  | Yes | 50 | 1.48 (0.58, 3.83) | 0.41 | 39 | 1.34 (0.48, 3.79) | 0.58 |
| Hypercholesterolemia | No | 527 |  |  | 397 |  |  |
|  | Yes | 275 | 0.74 (0.42, 1.31) | 0.3 | 236 | 0.6 (0.33, 1.12) | 0.11 |
| Abbreviations: OR = odds ratio; CI = confidence interval; GLR: Greater London Region, ER: Edinburgh region | | | | | | | |

**Supplementary Table 5** The ordinal logistic multivariate models for microbleeds (0, 1–3, 4+) (In population with **available APOE genotype data**) (continued)

|  |  |  | **Age Up to 70** |  |  | **Age 71 or older** |  |  | **Female** |  |  | **Male** |  |
| --- | --- | --- | --- | --- | --- | --- | --- | --- | --- | --- | --- | --- | --- |
| **With available APOE genotyping** |  | N | OR (95% CI), N=430 | P>\|z\| | N | OR (95% CI), N=372 | P>\|z\| | N | OR (95% CI), N=416 | P>\|z\| | N | OR (95% CI), N=386 | P>\|z\| |
| Age (continuous) |  |  | - | - |  | - | - | 416 | 1.05 (0.98, 1.13) | 0.16 | 386 | 1.06 (0.99, 1.13) | 0.11 |
| Sex | Female | 231 |  |  | 185 |  |  |  | - | - |  | - | - |
|  | Male | 199 | 1.31 (0.55, 3.15) | 0.54 | 187 | 1.59 (0.82, 3.09) | 0.17 |  | - | - |  | - | - |
| BMI (continuous) |  | 430 | 0.94 (0.84, 1.04) | 0.25 | 372 | 1 (0.92, 1.08) | 0.99 | 416 | 0.93 (0.85, 1.03) | 0.16 | 386 | 1.02 (0.93, 1.11) | 0.74 |
| Region of residence | GLR | 325 |  |  | 308 |  |  | 327 |  |  | 306 |  |  |
|  | ER | 105 | 0.38 (0.1, 1.37) | 0.14 | 64 | 0.4 (0.13, 1.21) | 0.11 | 89 | 0.4 (0.12, 1.39) | 0.15 | 80 | 0.33 (0.1, 1.04) | 0.06 |
| Education | Less than a bachelor’s degree | 179 |  |  | 177 |  |  | 196 |  |  | 160 |  |  |
|  | Bachelor's degree or higher | 251 | 0.5 (0.21, 1.22) | 0.13 | 195 | 0.55 (0.28, 1.05) | 0.07 | 220 | 0.68 (0.31, 1.47) | 0.32 | 226 | 0.46 (0.23, 0.94) | **0.03** |
| Amyloid | Non-elevated | 331 |  |  | 238 |  |  | 299 |  |  | 270 |  |  |
|  | Elevated | 99 | 2.5 (1.03, 6.07) | **0.04** | 134 | 0.94 (0.48, 1.85) | 0.87 | 117 | 2.06 (0.93, 4.58) | 0.08 | 116 | 1.05 (0.48, 2.27) | 0.91 |
| Hypertension | No | 291 |  |  | 192 |  |  | 265 |  |  | 218 |  |  |
|  | Yes | 139 | 1.11 (0.43, 2.87) | 0.83 | 180 | 1.5 (0.75, 2.98) | 0.25 | 151 | 1.94 (0.87, 4.33) | 0.11 | 168 | 1 (0.47, 2.1) | 0.99 |
| Type 2 Diabetes | No | 408 |  |  | 344 |  |  | 388 |  |  | 364 |  |  |
|  | Yes | 22 | 3.89 (0.67, 22.67) | 0.13 | 28 | 1.11 (0.35, 3.48) | 0.86 | 28 | 1.73 (0.43, 7.06) | 0.44 | 22 | 1.39 (0.37, 5.2) | 0.62 |
| Hypercholesterolemia | No | 306 |  |  | 221 |  |  | 295 |  |  | 232 |  |  |
|  | Yes | 124 | 0.36 (0.1, 1.26) | 0.11 | 151 | 0.89 (0.44, 1.79) | 0.75 | 121 | 1.09 (0.46, 2.58) | 0.85 | 154 | 0.63 (0.29, 1.38) | 0.25 |
| Abbreviations: OR = odds ratio; CI = confidence interval; GLR: Greater London Region, ER: Edinburgh region | | | | | | | | | | | | | |

**Supplementary Figure 1** Structural equation modelling (SEM) for the GLR (n =1,055).

In GLR participants, only hypertension, associated with age and sex, exhibited significance (Coef. 0.065) in relation to CMH positive groups. It highlights among females, being at the GLR site and having hypertension is connected to CMH presence, with the model fit indices NFI=0.62, CFI=0.60, RMSEA=0.10, AIC=276.1; NFI=0.94, CFI=0.96, RMSEA=0.04, and AIC=73.94 for the model excluding BMI, type 2 diabetes, and hypercholesteremia.


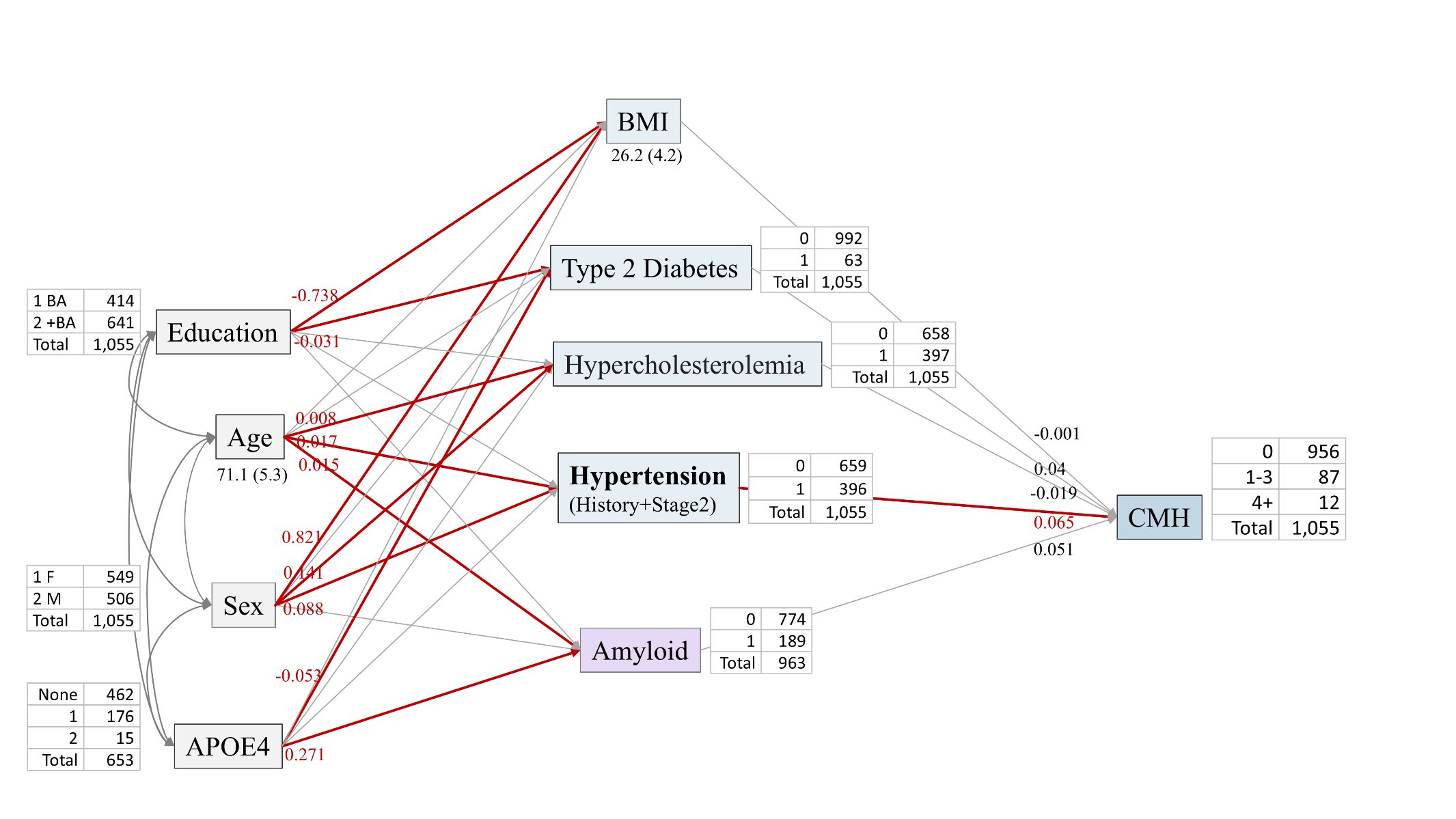


***Supplementary Figure 2*** Aβ and the ARWMC rating scale for regions of WMH in the presence of CMH (N=101)
(a) left and right parieto-occipital regions: AUROC curve 0.60 (95% CI: 0.50, 0.70)

(b) left and right frontal lobe regions: AUROC curve 0.63 (95% CI: 0.53, 0.73)

(a) (b)


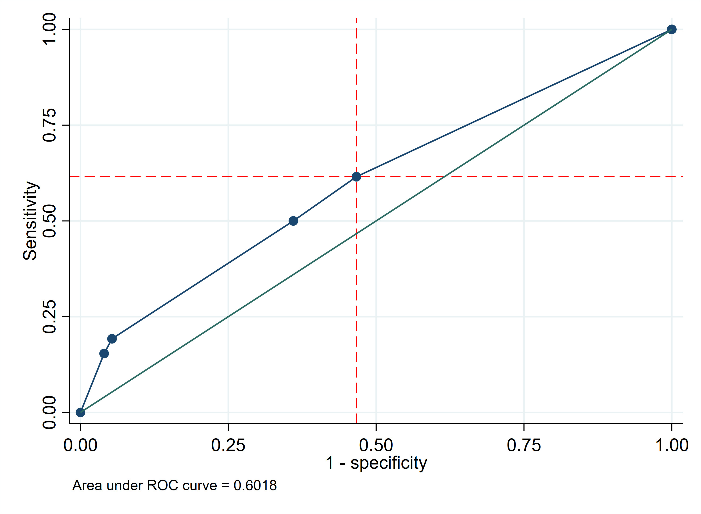

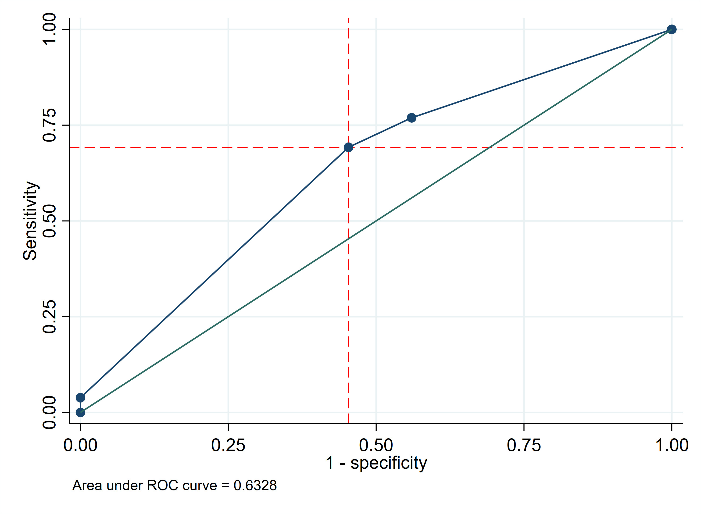


**Supplementary Figure 3** Incidental findings of other MRI abnormalities


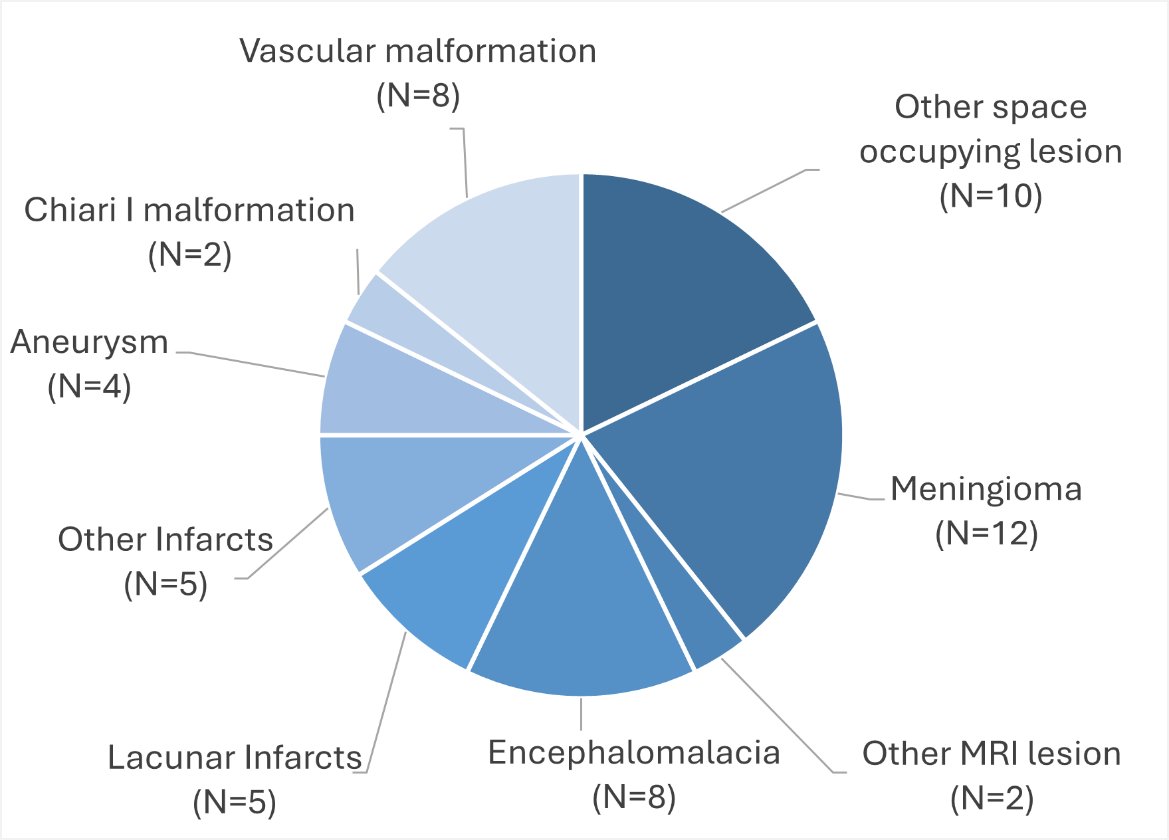


**Supplementary Text 1**

Evaluation of amyloid PET

PET scans were evaluated using a hybrid visual and quantitative approach. All exams were acquired in 3D mode with corrections for attenuation (CT-based), scatter and random coincidence. Visual reads were performed by one of three neuroradiologists in a central laboratory, according to the prescribing information for each tracer and blinded to SUVR. Quantitative analysis involved coregistration of the image to each participant’s baseline 3DT1 MRI. A composite SUVR was calculated as the volume-weighted average across FreeSurfer target and reference subregions derived from native-space MRI.

Positive SUVR thresholds for each tracer were as follows: Florbetapir: > 1.14 (whole Cb reference), Florbetaben: > 1.20 (whole Cb reference) and Flutemetamol: > 1.21 (whole Cb reference). In cases where visual and quantitative analysis gave discordant results, a scan with an above threshold SUVR was always classified as positive, provided it passed quality control (QC) and a positive primary visual read was classified as positive, if a second reader agreed.
